# Maintaining the Human Voice in Scientific Writing in the Era of Artificial Intelligence

**DOI:** 10.31662/jmaj.2025-0517

**Published:** 2026-02-13

**Authors:** Shigeki Matsubara

**Affiliations:** 1 Department of Obstetrics and Gynecology, Jichi Medical University, Tochigi, Japan; 2 Department of Obstetrics and Gynecology, Koga Red Cross Hospital, Koga, Ibaraki, Japan; 3 Medical Examination Center, Ibaraki Western Medical Center, Chikusei, Japan

**Keywords:** artificial intelligence, ChatGPT, paper, regulation, writing

## To the Editors,

The *JMA Journal* requires authors to disclose any artificial intelligence (AI) use, including manuscript generation ^(1)^. I wish the *JMA Journal*, at least for the time being, would maintain such AI regulation. This is because maintaining the human voice in scientific writing is increasingly important in the AI era.

Recently, some researchers claimed that the declaration of AI use in writing should not be mandatory ^(2)^. Several leading journals formally stated that AI may be freely used to edit manuscripts and need not be declared ^(3)^. Some journals, exhausted by policing authorship, are likely to adopt this stance. I appreciate that struggle―yet is it really necessary to declare this formally, making this the norm so soon?

Officially stating, “AI use is permitted without declaration,” may lead to unintended consequences: a considerable fraction of authors may take this as a recommendation―much like using a spellchecker. Some may feel compelled to use AI at least once before submission.

ChatGPT rarely responds, “no change required,” and almost always proposes modifications, even to well-written sentences. Experienced authors may resist, but many, especially non-native speakers, may not. Such unintentional AI intervention can thus spread silently and gradually, eroding diversity, rhythm, and personal tone that the original manuscript might have involved. Yet writing is not merely technical; it reflects thinking, judgment, and individuality. If manuscripts begin to sound uniform ^(4)^, readers lose the joy of reading, and authors lose the joy of writing ^(4), (5)^.

The current, somewhat ambiguous situation allows each author and journal to decide independently. Indeed, I have never heard of a study retracted solely for undeclared AI use. Discussion continues, yet the publication world already moves spontaneously, guided by experience, reason, and balance―much like common law. An open declaration permitting AI use may sound progressive, but it risks altering the atmosphere of publication, turning freedom of choice into a mild obligation.

Let the world continue under the current, functional ambiguity until AI use is proved completely safe. Too-early certainty may damage what ambiguity has traditionally protected: the heterogeneity, individuality, and warmth of scientific writing.

Having lived in medical writing for 47 years, I now find, at the sunset of my writing, that this ground-breaking issue has arisen. I have established my own style: AI hardly affects it. I worry for the next generations: Where will medical publication go? Can we continue to embrace medical writing in the future?

## Article Information

### Acknowledgments

A part of this concept was previously described and is cited.

### Author Contributions

Shigeki Matsubara designed this study, wrote, edited, and approved the final manuscript, and meets the ICMJE criteria for authorship.

### Conflicts of Interest

None

### Ethics Approval

Jichi Medical University does not demand institutional review board approval for this type of study. Patient anonymity preservation and informed consent for reporting are not applicable.

### Data Availability Statement

Data sharing is not applicable to this article because no new data were created or analyzed in this study.
